# Targeting Pyroptosis: New Insights into the Treatment of Diabetic Microvascular Complications

**DOI:** 10.1155/2022/5277673

**Published:** 2022-09-27

**Authors:** Junling Gu, Kang Geng, Man Guo, Wei Huang, Tingting Zhao, Xuling Li, You-Hua Xu, Yong Xu

**Affiliations:** ^1^Faculty of Chinese Medicine, State Key Laboratory of Quality Research in Chinese Medicine, Macau University of Science and Technology, Avenida Wai Long, Taipa, China; ^2^Department of Endocrinology and Metabolism, Metabolic Vascular Disease Key Laboratory of Sichuan Province, Sichuan Clinical Research Center for Nephropathy, Cardiovascular and Metabolic Diseases Key Laboratory of Luzhou, Luzhou, Sichuan, China; ^3^Department of Endocrinology, The Second People's Hospital of Yibin City-West China Yibin Hospital, Sichuan Univerity, Yibin, Sichuan, China; ^4^Department of Plastic and Burn Surgery, National Key Clinical Construction Specialty, The Affiliated Hospital of Southwest Medical University, Luzhou, Sichuan, China

## Abstract

Pyroptosis is an inflammatory form of programmed cell death that is dependent on inflammatory caspases, leading to the cleavage of gasdermin D (GSDMD) and increased secretion of interleukin (IL)-1*β* and IL-18. Recent studies have reported that hyperglycemia-induced cellular stress stimulates pyroptosis, and different signaling pathways have been shown to play crucial roles in regulating pyroptosis. This review summarized and discussed the molecular mechanisms, regulation, and cellular effects of pyroptosis in diabetic microvascular complications, such as diabetic nephropathy, diabetic retinopathy, and diabetic cardiomyopathy. In addition, this review aimed to provide new insights into identifying better treatments for diabetic microvascular complications.

## 1. Introduction

Diabetes is a low-grade inflammatory disease that seriously affects the quality of life of 463 million adults aged 20–79 years [1]. Diabetic microvascular complications (DMCs), such as diabetic nephropathy (DN), diabetic retinopathy (DR), and diabetic cardiomyopathy (DC), are the main contributors to the morbidity and mortality associated with diabetes. However, the pathogenesis and pathophysiology of these DMCs are complex. Chronic inflammatory response induced by hyperglycemia is a common mechanisms in DMCs. Further investigation of novel molecular mechanisms is required to develop new therapeutic approaches for DMCs.

Pyroptosis is a type of programmed cell death and its morphological characteristics are mainly caused by cell membrane rupture, chromatin condensation, nuclear fragmentation, and substantial release of cellular contents, including interleukin (IL)-1*β*, IL-18, and lactate dehydrogenase [2]. Pyroptosis is initially considered an innate immune and inflammatory response to invasion by external pathogens. Appropriate cell pyroptosis eliminates pathogens when they invade, but subsequent investigations suggest that pyroptosis is a double-edged sword, and that excess pyroptosis leads to a constant inflammatory response [3]. Recent studies have reported that a series of chronic inflammatory diseases, such as cardiovascular disease [4], rheumatoid arthritis [5], and nervous system diseases [6], are associated with pyroptosis, and that blocking excessive pyroptosis is a promising strategy for delaying disease development. Evidence suggests that diabetes is closely associated with pyroptosis [7]. This review provided an overview of the role of pyroptosis in DMCs and the effects of inhibition of the pyroptosis pathway in DMCs.

## 2. Pyroptosis and Pyroptosis-Related Mechanisms

tZychlinsky et al. first discovered the phenomenon of pyroptosis in 1992, which was initially considered apoptosis in macrophages infected by *Shigella flexneri* [8]. Studies have shown that pyroptosis is caused by caspase-1-regulated etiology. Scholars called this the canonical pyroptosis pathway and named it “pyroptosis” in 2001 to contrast with other types of cell death, meaning “fire” [9]. Thereafter, a noncanonical pyroptosis pathway was observed [10]. In 2015, Kayagaki and Shi demonstrated that gasdermin D (GSDMD) was the ultimate executor of pyroptosis [11, 12]. GSDMD is a gasdermin family member that can be cleaved into GSDMD-N fragments by inflammatory caspases, including caspase-1 and caspase-4/caspase-5/caspase-11 (caspase-4/caspase-5 in humans and caspase-11 in mice) [13]. The GSDMD-N fragment of ring-shaped oligomers anchored on the cell membrane forms pores to induce cell death [14].

**Figure 1:**
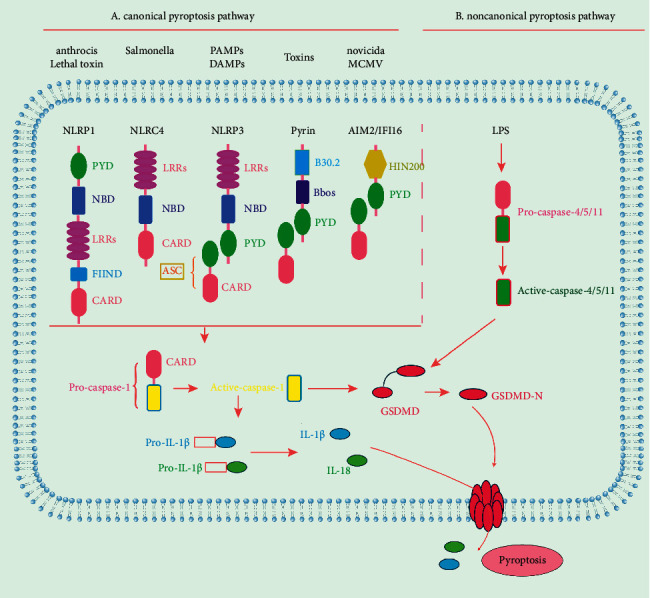
Canonical and noncanonical pathway pyroptosis. Pyroptosis mechanisms include canonical and noncanonical pathways. The left side of (a) shows the canonical pyroptosis pathway, which is caspase-1 dependent; the right side of (b) shows the noncanonical pathway, which is caspase-4/caspase-5/caspase-11 dependent; the cleavage of gasdermin D (GSDMD) (GSDMD-N domain) forms pores on the cell membrane, inducing pyroptosis.

Inflammation is a complex process that reflects local and systemic responses to different immunological and nonimmunological stimuli. Pyroptosis, which is essentially an inflammatory reaction, has provided a new perspective for research on the pathophysiological mechanisms of DMCs. Pyroptosis mechanisms include canonical and noncanonical pathways. The difference between the two pathways is primarily due to their triggering factors. The two pathways are independently discussed below, and the details can be found in Figures 1(a) and 1(b).

### 2.1. Canonical Pyroptosis Pathway

The canonical pyroptosis pathway is caspase-1-dependent and is triggered by inflammasomes [15]. In this pathway, pattern recognition receptors including Nod-like receptor (NLR) proteins (NLRP1, NLRP3, NAIP/NLRC4), pyrin, and ALR proteins (AIM2 and IFI16) as sensors, first recognize pathogen-associated molecular patterns (such as bacteria, viruses, and DNA damage) or danger-associated molecular patterns (such as uric acid and extracellular adenosine 5′‐triphosphate) [16]. During this process, NLRP1 and NLRC4 directly recruit pro-caspase-1, while binding between pro-caspase-1 and NLRP3/pyrin/ALR, and the activation of caspase-1 must be accompanied by the adaptor protein ASC to form inflammasomes [17]. Thereafter, pro-IL-1*β* and pro-IL-18 are sheared to activate IL-1*β* and IL-18, respectively, by activating caspase-1, and GSDMD is cleaved into GSDMD-N to form pores on the cell membrane [18].

### 2.2. Noncanonical Pyroptosis Pathway

The noncanonical pyroptosis pathway is caspase-11- or caspase-4/caspase-5-dependent (caspase-11 in mice and caspase-4/5 in humans) [19]. In 2011, Kayagaki et al. reported that the secretion of IL-1*β* from macrophages was inhibited in Casp11^−/−^ C57BL/6 or Casp1^−/−^ Casp11^129mt/129mt^ mice infected with Gram-negative bacteria and that cell death induced by caspase-11 activation was independent of NLRP3 and ASC [20]. Therefore, they proposed this phenomenon as the noncanonical inflammasome-triggered caspase-11 or the noncanonical pyroptosis pathway [20]. The study has shown that lipopolysaccharide (LPS) initially activates the non-canonical pyroptosis pathway, and this process is independent of Toll-like receptor 4 (TLR4) [21]. Lipid A in LPS binds to caspase-11/caspase-4/caspase-5 through the CARD-CARD domain. Subsequently, inflammatory caspases are activated, and GSDMD is cleaved to GSDMD-N to induce cell death [22]. Notably, although activated caspase-11/caspase-4/caspase-5 can induce pyroptosis, they cannot directly cleave pro-inflammatory cytokines [23].

## 3. Pyroptosis in Diabetic Microvascular Diseases

Recently, although limited, studies have attempted to explore the relationship between pyroptosis and DMCs and their potential mechanisms. Studies on pyroptosis and its associated diseases are summarized in this review.

### 3.1. Pyroptosis in Diabetic Nephropathy

DN is one of the most prevalent and serious microvascular complications associated with diabetes. Its early pathological characteristics include basement membrane thickening, increased mesangial matrix production, and extracellular matrix accumulation, with the subsequent development of glomerulosclerosis and tubulointerstitial fibrosis, eventually leading to proteinuria and irreversible renal damage. The early clinical diagnosis of DN is based on canonical biochemical markers, such as glomerular filtration rate, urinary microalbumin, urinary microalbumin to urinary creatinine ratio, serum creatinine, urinary cystatin C, and serum *β*2 microglobulin. In addition, some biomarkers related to the pathogenesis of DN, such as kidney injury molecule 1 (Kim-1), neutrophil gelatinase-associated lipocalin (NGAL), tissue inhibitor of metalloproteinases-2, insulin-like growth factor-binding protein 7 (IGFBP-7), vascular endothelial growth factor (VEGF), transforming growth factor-*β* (TGF-*β*), monocyte chemoattractant protein-1(MCP-1), and inflammatory cytokines, such as tumor necrosis factor (TNF)-*α*, MCP-1, and ILs (IL-1*α*, IL-1*β*, IL-18, IL-10), have also attracted significant attention [24, 25]. However, the pathogenesis of DN remains unclear, and treatment strategies are limited. Recent studies have found that pyroptosis is involved in pathophysiological processes and may be a potential therapeutic target.

#### 3.1.1. Glomerular Endothelial Cells

Glomerular endothelial cells (GECs) are located within the glomerulus and are the first layer to be involved in glomerular filtration. Recent findings suggest that both cell loss and inflammation in GECs are important causes of DN [26], and that pyroptosis may be a pivotal link between them. Activation of NLPR3 inflammasomes has been observed in the glomeruli of patients with DN and in animal models [27]. An *in vitro* study has reported that LPS directly induces pyroptosis in vascular endothelial cells [28]. Because low-grade inflammation and hyperglycemia form a vicious circle that promotes the development of DN toward end-stage renal disease, inhibiting pyroptosis may be an ideal strategy to block it. Our previous study showed that high glucose levels could induce pyroptosis in GECs, which was alleviated by a caspase-1 inhibitor or sodium butyrate [29]. Another study reported that hirudin ameliorated DN by inhibiting GSDMD-mediated pyroptosis in GECs [30].

#### 3.1.2. Podocytes

Podocytes are the outer glomerular filtration barriers. Podocyte fusion and foot process effacement cause proteinuria, and cell death and inflammation are the underlying mechanisms [31]. Podocyte pyroptosis has gradually been elaborated upon. Previous studies have demonstrated the activation of NLRP3/caspase-1/IL-1*β* in podocytes derived from patients with DN, db/db mice, and streptozotocin (STZ)-induced mice/rats [27, 32]. High glucose [27], D-ribose [33], and visfatin [34] levels directly induce the activation and release of NLRP3/ASC/caspase-1/IL-1*β* in podocytes, whereas inhibition or knockdown [33] of NLRP3 [32], ASC [33], and caspase-1 [33] improves the function of podocytes. A previous study showed that inhibition of NLRP3 reduced the expression of podocin and ameliorated renal fibrosis [32]. Another study reported that the inhibition of caspase-1/IL-18 signaling in DN could reduce albuminuria [35]. Additionally, high glucose levels activate caspase-11/caspase-4 and GSDMD-mediated pyroptosis, resulting in podocyte loss and DN development [36]. Thus, the intervention of the podocyte pyroptosis pathway may be a new target for the treatment of DN. Given the targets of pyroptosis, the regulatory mechanism of the pyroptosis pathway is gradually being studied. TLR4 knockdown attenuates high glucose-induced podocyte injury via the NALP3/ASC/caspase-1 signaling pathway [37]. There is also an indication that thioredoxin interaction protein (TXNIP) is involved in activating the high-glucose-induced NALP3 inflammasome and podocyte injury [38]. Forkhead box protein M1 transcriptionally activates sirtuin 4 and inhibits nuclear factor kappa B (NF-*κ*B) signaling and NLRP3 inflammasome to alleviate kidney injury and podocyte pyroptosis in DN [39]. Moreover, sublytic complement C5b-9 induces pyroptosis in podocytes via the KCNQ1 overlapping transcript 1 (KCNQ1OT1)/miR-486a-3p/NLRP3 regulatory axis [40]. A recent study by Ding et al. showed that MiR-21-5p in macrophage-derived extracellular vesicles could regulate pyroptosis-mediated podocyte injury induced by A20 in DN [41]. Furthermore, unexpectedly, some drugs have been found to exert a protective effect against DN via an antipyroptosis mechanism. Geniposide inhibits pyroptosis via the AMPK/SIRT1/NF-*κ*B pathway in podocytes in DN [42]. Atorvastatin protects podocytes via the metastasis-associated lung adenocarcinoma transcript 1 (MALAT1)/miR-200c/nuclear factor-erythroid 2-related factor 2 (NRF2) signaling pathway from hyperglycemia (HG)-induced pyroptosis and oxidative stress [43]. Catalpol effectively inhibits oxidative stress and inflammation accompanied by pyroptosis in podocytes via the AMPK/SIRT1/NF-*κ*B pathway [44]. The total flavones of *Abelmoschus manihot* (TFA) alleviate podocyte pyroptosis and injury by adjusting methyltransferase-like protein 3 (METTL3)-dependent m6A modification and regulating NLRP3-inflammasome activation and phosphatase and tensin homolog (PTEN)/phosphoinositide 3-kinase/protein kinase B (Akt) signaling [45]. As podocytes act as the last barrier against proteinuria, exploring agents that prevent damage to these cells is of special significance.

#### 3.1.3. Glomerular Mesangial Cell

Glomerular mesangial cells (GMCs) play a pivotal role in maintaining the basic structure of the glomerulus. Increasing the mesangial matrix and GMCs proliferation promotes DN development. Inflammation is an important factor in GMC proliferation [46]. Previous studies have shown that high glucose level significantly induces the expression of pyroptosis markers, including NLRP3, caspase-1, pro-caspase-1, IL-1*β*, and pro-IL-1*β*, in GMCs [47, 48], whereas inhibiting NLRP3 with MCC950 suppresses NLRP3/caspase-1/IL-1*β* activation and decreases renal fibrosis [32]. Nuclear enriched abundant transcript 1 and its target gene miR-34c are also found to regulate pyroptosis by mediating NLRP3 on GMCs in DN [49]. Furthermore, naringin and ginsenoside compound K have been reported to exert potential effects against pyroptosis in GMCs [50, 51].

#### 3.1.4. Renal Tubular Epithelial Cells

Renal tubular epithelial cells (RTECs) are more vulnerable to death because they are inevitably stimulated by various pathogenic factors, such as toxins, hypoxia, and metabolic disorders. In patients with diabetes, the expression of IL-18, an inflammatory cytokine released during pyroptosis in renal tubular cells, increases significantly (approximately 83%) [52], suggesting the potential involvement of pyroptosis in RTEC damage. The expression of NLRP3, caspase-1, and IL-1*β* increases significantly in both STZ-induced DN rats and high glucose-treated RTECs [53]. Inhibition of caspase-1 or GSDMD knockdown ameliorates RTEC pyroptosis and reduces kidney damage *in vivo* and *in vitro* [54, 55]. Moreover, upregulation of miR-23c inhibits RTEC pyroptosis by modulating MALAT1/ELAVL [53]. The TLR4/NF-*κ*B signaling pathway also modulates GSDMD-mediated pyroptosis in RTECs [56]. Recent studies have reported that antisense noncoding RNA in the INK4 locus/miR-497/TXNIP and KCNQ1OT1/miR-506-3p are involved in the regulation of high glucose-activated HK2 cell pyroptosis [57, 58]. Moreover, circACTR2 regulates high glucose-induced pyroptosis, inflammation, and fibrosis in proximal tubular cells [59]. Specific inhibitors of pyroptosis in RTEC have not been well studied, and existing evidence suggests a potential function for an A1 adenosine receptor agonist [35] or hirudin [30]. In summary, the canonical pyroptosis pathway is associated with intrinsic damage to RTECs and plays an essential role in DN development. RTEC pyroptosis inhibition may be a novel target for ameliorating albuminuria and renal fibrosis.

### 3.2. Pyroptosis in Diabetic Retinopathy

DR is a hallmark complication of diabetes and a leading cause of vision loss in adults. Loss of retinal pericytes is one of the earliest changes associated with DR, and it has been postulated to initiate or trigger microaneurysm formation, abnormal leakage, edema, and ischemia, provoking proliferative neovascularization in the retina. Although the pathophysiological mechanisms of DR are complex, vascular endothelial damage, increased vascular permeability, and neovascularization are the most common phenomena.

Hemoglobin A1c (HbA1c) is the only validated systemic biomarker for DR progression. However, only 6.6% of the variation in the risk of DR is explained by HbA1c levels [60]. In addition, there are some biomarkers for the pathogenesis of DR, including VEGF, pigment epithelium-derived factor, platelet-derived growth factor subunit B, photoreceptor-secreted retinol-binding protein 3, forkhead box protein O1, NRF2, atypical protein kinase C, and inflammatory cytokines, such as TNF-*α*, IL1*β*, IL-6, IL-8, chemokines, C-C motif ligand-2, intercellular cell adhesion molecule-1, and vascular cell adhesion molecule-1 [61]. Furthermore, long noncoding RNAs (lncRNAs) and circular RNAs (circRNAs) in whole blood can serve as novel non-invasive biomarkers for proliferative DR [62, 63]. However, there remains an urgent need to identify novel biomarkers for DR screening and detection.

Hyperglycemia-induced cell pyroptosis in the retina has been well demonstrated [64]. The density of corneal endothelial cells reduces and the expression of NLRP3, caspase-1, and IL-1*β* increases in patients with DR [65]. A similar event has been reported in STZ-induced diabetic rats [66]. Further studies have demonstrated that inhibiting caspase-1 [67] or silencing NLRP3 can reduce the secretion of caspase-1 and IL-1*β*, improve retinal layer thickness, and ameliorate blood-retinal barrier permeability [68], thus effectively retarding the progression of DR [69]. Moreover, the reactive oxygen species (ROS)/TXNIP/NLRP3 pathway is responsible for high glucose-induced retinal vascular permeability and pyroptosis in human retinal microvascular endothelial cells [69]. Further investigations have suggested that miR-590-3p induces the upregulation of NLRP1/NADPH oxidase 4, which participates in the above modulation [70]. Furthermore, miR-214 and KCNQ1OT1 are involved in the caspase-1 signaling pathway [65]. In addition, high glucose levels can induce pyroptosis in retinal pigment epithelial cells through the METTL3/miR-25-3p/PTEN/Akt signaling cascade or by regulating CircZNF532/miR-20b-5p/signal transducer and activator of transcription 3 [71, 72].

Although inhibition of caspase-1 or NLRP3 has been suggested to delay DR progression, a series of issues remain to be solved. First, current studies have mainly focused on the NLRP3-caspase-1 signaling pathway in DR. Therefore, the role of the noncanonical pyroptosis pathway in this disease remains unknown. Moreover, as inflammasome activation does not always trigger pyroptosis, other signaling pathways involved in pyroptosis should be investigated. Second, the regulatory mechanism of pyroptosis is currently focused on noncoding RNA, and other regulatory mechanisms are yet to be defined. Third, GSDMD is the ultimate executor protein in pyroptosis, and its function in DR requires further in-depth study. In addition, although studies have indicated the influence of resolvin D1, minocycline, caspase 1, and NLRP3 inhibitors (such as Mcc950), or H3 relaxin [73] on blocking the NLRP3-caspase-1 signaling pathway [65–67, 69], these have not been applied clinically, and more inhibitors are waiting to be screened.

### 3.3. Pyroptosis in Diabetic Cardiomyopathy

DC is the main cardiovascular complication that occurs in approximately 60% of patients with well-controlled diabetes, resulting in systolic and diastolic dysfunctions, which are independent risk factors for any vascular disease or hypertension [74]. Increasing evidence suggests that hyperglycemia, lipotoxicity, and mitochondrial uncoupling contribute to cardiac inflammation, which plays an important role in the pathogenesis and progression of DC [75]. In this process, cytoplasmic calcium is increased, triggering mitochondrial changes, the production of ROS is increased, and activating ROS levels lead to oxidative damage in DC, among which oxidative stress and chronic inflammation are critical [75].

A broad spectrum of cardiovascular biomarkers that have been described in patients with DC includes brain natriuretic peptide (BNP), cardiac troponins (T, N, and I), and matrix metalloproteinases (MMPs), particularly MMP-9. Some biomarkers related to the pathogenesis of DR have been reported, such as cardiotrophin-1, IGFBP7, TGF-*β*, activin A, ROS-induced inflammatory cytokines (TNF-*α*, IL-6), galectin-3, suppression of tumorigenicity 2 (sST2), lncRNAs, and microRNAs [76]. However, the predictive roles of these biomarkers in patients with DM remain unclear owing to limited evidence [77]. We hope to identify novel biomarkers for DC screening.

Due to the undefined pathophysiology of DC, several studies have attempted to investigate the role of pyroptosis in DC. In early 2014, Luo et al. [78] reported that the expressions of NLRP3, ASC, caspase 1, and IL-1*β* increased significantly in cardiomyocytes of STZ-treated diabetic rats and that NLRP3 silencing or inhibition of caspase-1 reduced their expression, alleviated left ventricular dysfunction, and ultimately reversed myocardial remodeling in DC. Similar findings were observed in a DC model developed from C57BL/6J mice [79, 80]. Apart from the caspase-1 modulated pathway, noncoding RNAs are also involved in this process. Upregulation of microRNA-30d directly modulates the downregulation of forkhead box class O 3a in STZ-treated rats or high glucose-induced cardiomyocytes [81]. The study has observed that knockdown of the long noncoding RNA KCNQ1OT1 inhibits high glucose-induced pyroptosis by upregulating miR-214-3p and reducing caspase-1 expression in AC16 cells and primary cardiomyocytes [82]. Similarly, another study suggested that lncRNA-MALAT1 targeted miR-141-3p to promote HG-induced H9C2 cardiomyocyte pyroptosis [83]. Moreover, activation of the transforming growth TGF-*β*1/Smads pathway in cardiac fibroblasts is repressed by KCNQ1OT1 knockdown [80]. A further study reported that hsa_circ_0076631, a caspase-1 related circRNA that suppresses miR-214-3p, increased in high-glucose-treated cardiomyocytes or serum from patients with diabetic [84]. Other noncoding RNAs, including AIM2 [85], miR-9 [86], GAS5 [87], and MIAT [88], are also potential modulators of pyroptosis in DC. A recent study demonstrated that the circRNAs circ_0071269 might promote the development of DC through the miR-145/GSDMA axis [89]. In addition, overexpression of mitochondrial aldehyde dehydrogenase 2 can reduce the high glucose-induced occurrence of pyroptosis in H9C2 cardiac cells [90].

Many drugs have been demonstrated to have some effects against pyroptosis (e.g., metformin [91], exendin-4 [92], pyrroloquinoline quinone (PQQ) [93], and skimming) [94]. A natural coumarin derivative has been found to protect against experimental DC by inhibiting pyroptosis in cardiomyocytes [94]. Empagliflozin has also been confirmed to alleviate the activation of NLRP3 and subsequent cardiomyocyte pyroptosis in the diabetic heart [95]. Overall, these studies indicate that the role of pyroptosis in DC and inhibition of the pyroptosis pathway might have an advantage in their ability to protect DC.

## 4. Conclusions and Prospects

In conclusion, the pathophysiological mechanisms underlying DMCs are complex and unclear. However, pyroptosis, a type of programmed cell death that triggers inflammatory reactions, provides a novel perspective for determining the mechanisms and therapeutic targets of DMCs (Table 1). These experiments have demonstrated the involvement of pyroptosis in the pathophysiological process of DMCs *in vitro* and *in vivo*. Based on an enhanced understanding of these existing results, we further explore how pyroptosis and inflammasome are activated, including some novel studies related to pyroptosis regulation and its relationship with DMCs, to provide a promising avenue for DMC prevention and treatment. Three new insights have been discovered regarding the regulation of pyroptosis in DMCs:

1. The important causes of DMCs are oxidative stress and inflammation resulting in pyroptosis. Based on the antioxidative stress and antiinflammatory strategies, we determined effective drugs or small-molecule compounds against pyroptosis to improve DMCs and further explored the regulation of pyroptosis and its potential mechanisms, which will open up a new direction for the prevention and treatment of DMCs.
2. The pro-inflammatory effect of pyroptosis is a key factor in the development of DMCs. Some studies have found that several upstream regulatory signaling proteins, noncoding RNAs, and circRNAs play a role in the upregulation of pyroptosis. Furthermore, autophagy plays a protective role in the inhibition of NLPR3 overactivation [96]. Therefore, further clarification of the regulatory mechanism of pyroptosis and targeting these pathways will also be an effective means to prevent and treat DMCs.
3. The inflammatory response is an important factor in the development of DMCs. Pyroptosis maintains or expands inflammation by tightly controlling the inflammatory response via the release of IL-1*β* and IL-18. IL-1*β* and IL-18 are two essential pro-inflammatory cytokines that are upregulated in tissue-resident cells of patients and animals with diabetes and are further induced by positive feedback to pyroptosis to form inflammatory storms [97]. Based on the perspective of the upregulation of different pro-inflammatory factors triggered by metabolic and hemodynamic disorders, controlling the chronic inflammatory microenvironment of diabetes will provide potential insights into the prevention and treatment of DMCs.

Nevertheless, whether some promising pyroptosis biomarkers can be used as novel targets for diagnosing and treating DMCs requires further investigation, particularly in animal experiments and clinical studies. Further investigations into the exact molecular and regulatory mechanisms of pyroptosis are necessary for DMCs. Furthermore, GSDMD, as the ultimate pyroptosis protein, should be investigated for its function in DMCs and to determine whether inhibiting GSDMD is a more valuable therapeutic target for these complications. Moreover, inhibition or activation of the pyroptosis pathway affects various aspects of physiology and can have different effects. It is necessary to consider the overall body balance when studying drugs that target the pyroptosis pathway in DMCs.

## Figures and Tables

**Table 1:** Summary of the effects of different methods or drugs on pyroptosis *in vivo* and *in vitro*.

Methods/drugs	Intervention targets	Cell type/animal model	Effects
Ac-YVAD-CMK [29, 33]	Caspase-1 inhibitor	GECs [29]	Reduce the expression of NLRP3, caspase-1, and IL-1*β*
VX-765 [51]		Immortalized mouse podocyte cell line [29, 33]	
		HBZY-1 [51]	
Sodium butyrate [29]	Caspase-1-GSDMD,	GECs	Ameliorate caspase-1-GSDMD-IL-1*β*/IL-18 canonical pyroptosis pathway and NF-*κ*B/I*κ*B-*α* signaling pathway
	NF-*κ*B/I*κ*B-*α*		
Hirudin [30]	IRF2-GSDMD	GECs, RTECs, and BMDMs	Inhibit IRF2-induced GSDMD
MCC950 [32]	NLRP3 inhibitor	db/db mice and rat mesangial cells<	Inhibit the NLRP3/caspase-1/IL-1*β* pathway
Knockdown TLR4 [37]	NALP3/ASC/caspase-1	Mouse podocytes	Inhibit NALP3/ASC/caspase-1 signaling pathway
Knockdown TXNIP [38]	TXNIP	Immortalized human podocyte cell line	Inhibits HG-induced NALP3 inflammasome activation, IL-1*β* production, and podocyte injury
Overexpress FOXM1 [39]	SIRT4	MPC5 cells	Inhibits NF-*κ*B signaling and NLRP3/caspase-1
sC5b-9	NLRP3	MPC5 cells	Inhibit the expression of NLRP3, caspase-1, GSDMD-N, IL-1*β*, and IL-18
Knockdown KCNQ1OT1			
Upregulate			
MiR-486a-3p [40]			
miR-21-5p inhibitor [41]	miR-21-5p/A20	MPC5 cells	Inhibit the expression of NLRP3, caspase-1, and IL-1*β*
Geniposide [42]	APMK/SIRT1/NF-*κ*B	HFD/STZ-induced DN mice and podocyte	Activate APMK/SIRT1 pathway and downregulate the relative levels of p–NF–*κ*B, ASC, cleave-IL-1*β*, NLRP3, cleave-caspase-1, and GSDMD-N
Catalpol [44]			
Atorvastatin [43]	MALAT1/miR-200c/NRF2	MPC-5 cells	Reduce the expression of NLRP3, caspase-1, and GSDMD via the downregulation of MALAT1/miR-200c and promotion of the expression of NRF2
TFA [45]	METTL3-dependent m6A	MPC-5 cells	Downregulate the expression of GSDMD-N, IL-1*β*, and IL-18;
	PTEN/PI3K/Akt		Upregulated the protein expression levels of nephrin, ZO-1, WT1, and podocalyxin in podocytes
Naringin [50]	NLRP3	Rat mesangial cells	Reduce the expression of inflammatory factors via the NLRP3-caspase-1-IL-1*β*/IL-18 signaling pathway
Ginsenoside compound K [51]	NLRP3	HBZY-1	Inhibit ROS-mediated activation of NLRP3 inflammasome and NF-*κ*B/p38 signaling pathway
	NF-*κ*B/p38		
Downregulate MALAT1 [53]	ELAVL1	HK-2 cells	Inhibit the expression of MALAT1
Upregulate			Downregulate the expression of ELAVL1, NLRP3, caspase-1, and IL-1*β*
MiR-23c [53]			
TAK-242 [56]	TLR4 inhibitor	HK-2 cells	Reduce GSDMD-N-induced pyroptosis via the inhibition of TLR4/NF-*κ*B signaling
Downregulate KCNQ1OT1 [57, 65]	NLRP3	HK-2 cells [57]	Inhibits pyroptosis via the NLRP3/caspase-1/IL-1*β* signaling
Upregulate		Corneal endothelial cells [65]	
MiR-214 [65]			
Knockdown ANRIL [58]	TXNIP	HK-2 cells	Inhibit caspase-1-dependent pyroptosis
Upregulate miR-497 [58]			
Resolvin D1 (RvD1) [66]	NLRP3	STZ-induced diabetic retinopathy rats	Inhibit the activation of the NLRP3 inflammasome and downregulate the levels of NLRP3, ASC, cleaved-caspase-1, IL-1*β,* and IL-18
Upregulate	NLRP1 and NOX4	HRMECs	Suppress pyroptosis by targeting NLRP1 and inhibit the NOX4/ROS/TXNIP/NLRP3 pathway
MiR-590-3p [70]			
Overexpress METTL3 [71]	miR-25-3p/PTEN	ARPE-19	Increase p-Akt level and attenuate the expression of caspase-1, GSDMD, NLRP3, IL-1*β* and IL-18
Knockdown	STAT3	ARPE-19	Inhibit STAT3 expression and decrease the expression of pro-caspase-1, caspase-1, NLRP3, IL-1*β*, and IL-18
CircZNF532 [72]			
Upregulate miR-20b-5p [72]			
H3 relaxin [73]	P2X7R/NLRP3	HRMECs	Attenuate P2X7R-mediated NLRP3 inflammasome activation and the expression of caspase-1, GSDMD, IL-1*β*, and IL-18
Silence KCNQ1OT1 [80, 82]	TGF-*β*1/Smads/caspase-1 [80]	C57BL/6 mice [80, 82]	Alleviate pyroptosis by targeting miR-214-3p
Upregulate miR-214-3p [80, 82]	NLRP3/caspase-1 [82]	Cardiac fibroblasts of neonatal C57BL/6 mice [80]	
		AC16 cells and primary cardiomyocytes [82]	
Downregulate microRNA-30d [81]<	Foxo3a and ARC<	Rat cardiomyocytes	Inhibit pyroptosis via the promotion of the expression of Foxo3a and ARC
Knockdown MALAT1 [83]	miR-141-3p	H9c2 cells	Upregulate miR-141-3p and attenuate the expression of ASC, caspase-1, GSDMD, GSDMD-N, and NLRP3
Knockdown AIM2 [85]	AIM2	H9C2 cells	Alleviated GSDMD-N-related pyroptosis
MicroRNA-9 [86]	ELAVL1	Mouse macropage RAW 264.7 cells	Attenuate the expression of ELAVL1 and inhibit pyroptosis
Upregulate GAS5 [87]	miR-34b-3p/AHR	HL-1 cells	Repress NLRP3 inflammasome activation-mediated pyroptosis via the inhibition of miR-34b-3p and enhance the gene and protein expression of AHR
Silence	miR-214-3p/caspase-1	Mice cardiomyocytes	Repress caspase-1-mediated pyroptosis via the inhibition of miR-34b-3p
MIAT [88]			
Knockdown circ_0071269 [89]	miR-145/GSDMA	H9c2 cells	Inhibit pyroptosis via the downregulation of GSDMA
		STZ-induced DM mice	
Overexpress ALDH2 [90]	NLRP3	H9c2 cells	Inhibit NLRP3/caspase-1 dependent pyroptosis
Metformin [91]	NLRP3	STZ-induced C57BL/6 mice primary cardiomyocytes from neonatal mice	Inhibit the NLRP3 inflammasome via AMPK/mTOR-dependent effects
Exendin-4 [92]	ROS/pAMPK/TXNIP	Primary cardiomyocytes	Inhibit pyroptosis via the downregulation of TXNIP
		HFD-fed mouse model	
PQQ [93]	NF-*κ*B/NLRP3	AC16 cells	Decrease pyroptosis-related protein levels
		STZ-induced diabetic mice	
Skimmin [94]	NLRP3	Primary neonatal cardiomyocytes	Decrease the expression of NLRP3, caspase-1, and IL-1*β*
		STZ-induced diabetic rat	
Empagliflozin [95]	NLRP3	db/db mice	Alleviate the activation of NLRP3 inflammasome and reduce the expression of cleaved-caspase-1, IL-1*β*, and cleaved-GSDMD

NLRP3: the nucleotide-binding oligomerization domain-like receptor family pyrin domain-containing 3; IL: interleukin; GSDMD: gasdermin D; GSDMA: gasdermin A; GSDMD-N: gasdermin N-terminal; TXNIP: thioredoxin-interacting protein; NF-*κ*B: nuclear factor *κ*B; I*κ*B-*α*: nuclear factor of kappa light polypeptide gene enhancer in B-cells inhibitor, alpha; IRF2: interferon regulatory factor 2; MALAT1: metastasis-associated lung adenocarcinoma transcript; NRF2: nuclear factor-erythroid factor 2-related factor 2; LncRNA: long noncoding RNA; TLR4: toll-like receptors-4; ASC: apoptosis-associated speck-like protein containing a CARD; FOXM1: Forkhead box protein M1; SIRT4: sirtuin 4; sC5b-9: the terminal complement complex; RvD1: resolvin D1; TFA: the total flavones of Abelmoschus manihot; MCC950: an inhibitor of NLRP3; APMK: AMP-activated protein kinase; SIRT1: sirtuin 1; AIM2: absent In Melanoma 2; METTL3: methyltransferase-like protein 3; ZO-1: zonula occluden-1; WT1: Wilms tumor protein; p-Akt: phosphorylated Akt; STAT3: signal transducer and activator of transcription 3; P2X7R: the P2X purinoceptor 7; ELAVL1: ELAV-like protein 1; GAS5: growth arrest specific 5; AHR: aryl hydrocarbon receptor; MIAT: myocardial infarction associated transcript; ALDH2: mitochondrial aldehyde dehydrogenase 2; ALDH2: mitochondrial aldehyde dehydrogenase 2; PQQ: pyrroloquinoline quinone; GECs: glomerular endothelial cells; RTECs: renal tubular epithelial cells; BMDMs: bone-marrow-derived macrophages; HRMECs: human retinal microvascular endothelial cells; MPC5 cells: mouse podocyte cell line; HK-2 cells: human tubular cells; ARPE-19: the human retinal pigment epithelium (RPE) cell line; HL-1 cells: cardiac muscle cell line; H9c2 cells: the rat embryonic cardiomyocyte cell line; AC16 cells: human myocardial cells; HFD: high-fat diet; STZ: streptozotocin.

## Data Availability

No data were used to support this study.
